# Refractory occipital neuralgia treatment with nerve decompression surgery: a case series

**DOI:** 10.3389/fneur.2023.1284101

**Published:** 2023-11-28

**Authors:** William G. Austen, Katya Remy, Kathryn Packowski, Merel H. J. Hazewinkel, Lisa Gfrerer, Paul G. Mathew

**Affiliations:** ^1^Division of Plastic and Reconstructive Surgery, Massachusetts General Hospital, Boston, MA, United States; ^2^Division of Plastic and Reconstructive Surgery, Weill Cornell Medicine, New York, NY, United States; ^3^Department of Neurology, Mass General Brigham Health, Foxborough, MA, United States; ^4^Department of Neurology, Harvard Vanguard Medical Associates, Braintree, MA, United States

**Keywords:** occipital neuralgia, headache, migraine, pain, nerve decompression surgery, outcomes

## Abstract

**Background:**

The management of refractory occipital neuralgia (ON) can be challenging. Selection criteria for occipital nerve decompression surgery are not well defined in terms of clinical features and best preoperative medical management.

**Methods:**

In total, 15 patients diagnosed with ON by a board-certified, fellowship-trained headache specialist and referred to a plastic surgeon for nerve decompression surgery were prospectively enrolled. All subjects received trials of occipital nerve blocks (NB), at least three preventive medications, and onabotulinum toxin (BTX) prior to surgery before referral to a plastic surgeon. Treatment outcomes included headache frequency (headache days/month), intensity (0–10), duration (h), and response to medication/injectable therapies at 12 months postoperatively.

**Results:**

Preoperatively, median headache days/month was 30 (20–30), intensity 8 (8–10), and duration 24 h (12–24). Patients trialed 10 (±5.8) NB and 11.7 (±9) BTX cycles. Postoperatively, headache frequency was 5 (0–16) days/month (*p* < 0.01), intensity was 4 (0–6) (*p* < 0.01), and duration was 10 (0–24) h (*p* < 0.01). Median patient-reported percent resolution of ON headaches was 80% (70–85%). All patients reported improvement of comorbid headache disorders, most commonly migraine, and a reduction, discontinuation, or increased effectiveness of medications, NB and BTX.

**Conclusion:**

All patients who underwent treatment for refractory ON by a headache specialist and plastic surgeon benefited from nerve decompression surgery in various degrees. The collaborative selection criteria employed in this study may be replicable in clinical practice.

## Introduction

Occipital neuralgia (ON) is a disabling headache disorder that involves lancinating pain in the distribution of the greater occipital nerves (GON), lesser occipital nerves (LON), and/or third occipital nerves (TON). The GON is most frequently involved and presents with pain that originates in the neck or skull base and radiates superiorly toward the fronto-orbital regions (1). Less frequently, the LON is involved, often in conjunction with the involvement of the GON. ON involving the LON travels laterally in the occipital scalp and radiates toward the ipsilateral ear and temple (2). The TON is located medial and more caudally to the GON and innervates the upper neck and lower occipital scalp.

According to the International Classification of Headache Disorders Third Edition (ICHD-3) diagnostic criteria, ON involves pain of shooting, stabbing or sharp in quality, palpation tenderness in the distribution of the involved nerve(s), and improvement of symptoms with nerve blocks (NB) (3). The diagnostic criteria are listed in Table 1.

**Table 1:** International classification of headache disorders, 3 [r]^d^ edition, occipital neuralgia diagnostic criteria.

A. Unilateral or bilateral pain fulfilling criteria B-E
B. Pain is located in the distribution of the greater, lesser, and/or third occipital nerves
C. Pain has 2 of the following 3 characteristics:
1. Recurring in paroxysmal attacks lasting from a few seconds to minutes
2. Severe intensity
3. Shooting, stabbing, or sharp in quality
D. Pain is associated with both of the following:
1. Dysesthesia and/or allodynia apparent during innocuous stimulation of the scalp and/or hair
2. Either or both of the following:
a. Tenderness over the affected nerve branches
b. Trigger points at the emergence of the greater occipital nerve or in the distribution of C2
E. Pain is eased temporarily by local anesthetic block of the affected nerve
F. Not better accounted for by another International Classification of Headache Disorder-3 diagnosis.

In clinical practice, isolated ON as the sole chief complaint is uncommon, but ON can more commonly be appreciated in patients who have another headache disorder such as migraine (4). Consequently, ON tends to be underdiagnosed in clinical practice (5). In a prevalence study conducted at a headache specialty clinic, 25% of patients complaining of headaches were diagnosed with ON, and most of these patients also had another coexisting headache disorder (4). When ON coexists with other headache disorders, it can serve to either trigger or worsen other types of headaches. Therefore, inadequate treatment of ON will often result in the exacerbation of increased resistance to coexisting headache disorders, necessitating greater use of medications.

The treatment of ON is multimodal. Physical therapy including postural training can improve symptoms such as muscle tension but is often insufficient (6). Medication options span various pharmacologic classes including anti-inflammatories, anticonvulsants, tricyclic anti-depressants, selective norepinephrine reuptake inhibitors, muscle relaxants, and CGRP antagonists (7, 8). However, the use of medications for headaches has limitations in terms of contraindications, side effects, and inconsistent efficacy (9, 10). A response to NB is part of the ICHD-3 diagnostic criteria for ON and can provide therapeutic remissions lasting for days, weeks, months, or even years. In patients with longer-lasting benefits, it is hypothesized that larger volume NB through hydro-dynamic forces can cause an expansion of the muscle, fascia, and other surrounding tissues that may be compressing the occipital nerves (11, 12). Onabotulinum toxin A (BTX) has also demonstrated efficacy in treating ON (13, 14).

For patients with refractory ON, radiofrequency ablation (RFA), neurostimulation, and surgical nerve decompression are therapeutic options. Although RFA has demonstrated efficacy for the treatment of ON, improvement of pain is often temporary and complications include permanent iatrogenic nerve injury, which can result in reduced effectiveness of subsequent peripheral nerve sparring decompression procedures (15, 16). Implanted neurostimulation has also been shown to be able to effectively treat refractory ON and reduce medication use (17). However, the resolution of pain may only be temporary, and devices have technical limitations and are associated with complications such as electrode displacement, battery replacement, hardware malfunction, and infection (18).

Nerve decompression surgery is indicated in patients who have failed management with conservative therapies. The principles of occipital nerve decompression surgery include the release of affected occipital nerves at all possible compression points and cushioning of the nerve to avoid scarring (19–21). The occipital nerves can be compressed by fascia, scar, muscle, or vasculature along its trajectory. Most frequently, the occipital nerves are found to be compressed by a thickened overlying trapezius fascia, commonly seen in patients with previous head or neck injury (22). The symptomatic improvement of ON from nerve decompression surgery has been demonstrated in multiple studies (23–29). Although many patients can benefit from surgery, a detailed analysis revealed a binary distribution of outcomes, with most patients either improving completely or not at all after surgery (30). This highlights the importance of establishing the correct diagnosis and proper patient selection for nerve decompression surgery to ensure successful outcomes, and conversely, to prevent surgical treatment of patients who are unlikely to benefit from the procedure.

Currently, nerve decompression surgery is underrecognized as a possible treatment option for patients with refractory ON. Education regarding the diagnosis of ON and the role of surgical treatment is necessary to broaden and improve treatment algorithms for ON. The objective of this study is to describe the clinical features, treatment path, selection process, and outcomes of occipital nerve decompression surgery in patients with refractory ON under the care of both neurologists and plastic surgeons.

## Methods

Institutional review board approval was obtained at the Massachusetts General Hospital in Boston, Massachusetts, with all patients providing informed consent. This prospective case series includes 15 consecutive patients who were managed and referred by a single-board certified, fellowship-trained headache specialist, and neurologist (PGM) to a board-certified plastic surgeon (WGA) for ON treatment occipital nerve decompression surgery between 2015 and 2022. Patient selection criteria for nerve decompression surgery candidacy were based on five internal selection criteria formulated by the neurologist and plastic surgeon:

1. Diagnosis of ON based on the ICHD-3 criteria preferably by a headache specialist/neurologist.
2. At least 15 headache days/month with failure of at least three different oral preventative medications (e.g., anticonvulsants, tricyclic antidepressants, and selective norepinephrine reuptake inhibitors), large volume occipital NB (6 cc of 0.75% bupivacaine without steroids), and at least three cycles of BTX injections.
3. Investigation of other potential causes of ON-like headaches including cervical spine MRI and evaluation and management by a physical therapist who has expertise in cervical pathology including ON.
4. An identifiable trigger/pain point along the course of the GON, LON, and/or TON using pain sketches, demarcation by the patient with an index finger, tenderness/Tinel sign, +/- a positive Doppler, suggesting the site of anatomic compression evaluated by the surgeon.
5. A positive NB response was performed by the surgeon at the presumed site of compression. Nerve block response was defined as at least a 50% relative reduction of ON headache intensity with a duration of at least 24 h.

Following occipital nerve decompression surgery, the treatment outcome was evaluated at 12 months postoperatively in terms of headache frequency (headache days per month), intensity (scale of 0–10), and duration (h), as well as changes in medications, NB and BTX injections. Patients were also questioned about the percent-resolution of ON headaches following surgery based on ON headache frequency, intensity, and duration. Although ON typically involves lancinating pain lasting for seconds to minutes, during a flare, some patients will have volleys of pain, and others will have a baseline steady pain in the same nerve distribution as the ON with superimposed lancinating pain, that is why duration was measured in hours to account for these two common presentations of ON. Patients were also questioned about the effect of nerve decompression surgery on reducing coexisting headaches. Data were collected prospectively using REDCap questionnaires preoperatively and at 12 months postoperatively. Data quantifying the number of preoperative treatments (oral medications, NB, and BTX) were retrospectively collected from chart reviews.

Statistical analyses were conducted using SAS^®^ software (SAS Institute Inc., Cary, NC). Descriptive statistics of continuous variables were reported using means and standard deviations or median and interquartile range depending on normality. Frequencies and percentages were used for categorical variables. Paired *t*-tests were performed to compare preoperative and postoperative ON headache frequency, duration, and intensity. A *p* < 0.05 was considered statistically significant.

### Data availability

Anonymized data will be shared by request from any qualified investigator.

## Results

All 15 patients were included in the final analysis. Patients were predominantly female (80%). The average age of ON onset was 20 (±8) years. The time between the onset of ON headache and surgical decompression averaged at 19.9 (±13.6) years. Prior to nerve decompression surgery, median headache days per month was 30 (20–30), duration was 24 h (12–24), and intensity was 8 (8–10). ON was bilateral in 13 patients (86.7%) and unilateral in 2 patients (13.3%). All patients were diagnosed with at least one additional headache disorder. Most patients (9 patients, 60%) had coexisting chronic migraine in the form of holocephalic throbbing pain, difficulty concentrating, photophobia, phonophobia, and nausea during attacks. Other headache diagnoses included persistent posttraumatic headache in three patients (20%), new daily persistent headache (NDPH) with migrainous features in two patients (13.3%), episodic migraine in one patient (6.7%), and trigeminal neuralgia in one patient (6.7%). Twelve patients (80%) reported a history of head or neck injury prior to ON onset. Patient demographics and ON headache characteristics are presented in Tables 2, 3, respectively.

**Table 2:** Patient demographics and characteristics.

**Variable**	**Patients (*n* = 15)**
Age, years, mean (SD)^*^	39.9 (12.2)
BMI, kg/m^2^, mean (SD)^*^	28.7 (7.7)
Race, *n* (%)	12 (80)
White	12 (80)
Puerto Rican	3 (20)
Ethnicity, *n* (%)	
Not Hispanic or Latino	12 (80)
Hispanic or Latino	3 (20)
Gender, *n* (%)	
Female	12 (80)
Male	3 (20)
Headache diagnoses, *n* (%)
Occipital neuralgia	15 (100)
Chronic migraine	9 (60)
Post-traumatic headache	3 (20)
NDPH with migrainous features	2 (13.3)
Episodic migraine	1 (6.7)
Trigeminal neuralgia	1 (6.7)
Pain disorders, *n* (%)	15 (100)
TMJ dysfunction	10 (66.7)
Fibromyalgia	6 (40)
Shoulder/neck muscle pain	4 (26.7)
Cervical radiculopathy	1 (6.7)
Psychiatric disorders, *n* (%)	9 (60)
Depression	6 (40)
Anxiety	6 (40)
PTSD	2 (13.3)
Suicide attempt	1 (6.7)
History of head or neck injury, *n* (%)	12 (80)
Trauma	11 (73.3)
Viral meningitis	1 (6.7)
Family history of migraine, *n* (%)	
Yes	12 (80)
No	3 (20)
Work status, *n* (%)	
Full time	7 (46.7)
Part time	3 (20)
Disabled	4 (26.7)
On sick leave	1 (6.7)

^*^Age and BMI at the time of surgery.

NDPH, new daily persistent headache; ON, occipital neuralgia; IQR, interquartile range; SD, standard deviation.

**Table 3:** ON pain characteristics.

**Variable**	**Patients (*n* = 15)**
Age of ON onset, years, mean (SD)	20 (8)
Frequency, days per month, median (IQR)	30 (20–30)
Duration, hours, median (IQR)	24 (12–24)
Intensity, scale 1–10, median (IQR)	8 (8–10)
Laterality, *n* (%)	
Bilateral	13 (86.7)
Unilateral	2 (13.3)
Start location, *n* (%)	
Back of head	15 (100)
Radiation, *n* (%)	
Fronto-orbital region	12 (80)
Ears	7 (46.7)
Temples	6 (40)
Quality, *n* (%)	
Lancinating, electric, shooting, stabbing, sharp	15 (100)
Ache/pressure	11 (73.3)
Throbbing/pounding	9 (60)
Like a tight band	7 (46.7)
Constricting, squeezing	3 (20)
Dysesthesia and/or allodynia, *n* (%)	12 (80)
Associated symptoms, *n* (%)	
Difficulty concentrating	14 (93.3)
Light/noise sensitivity	13 (86.7)
Nausea	11 (73.3)
Tight neck muscles	10 (83.3)
Blurred/double vision	9 (60)
Dizziness	9 (60)
Light-headedness	8 (53.3)
Flashing or colored lights	7 (46.7)
Numbness/Tingling	7 (46.7)
Vomiting	4 (26.7)
Runny nose	4 (26.7)
Speech difficulty	4 (26.7)
Loss of vision	3 (20)
Eyelid puffy	3 (20)
Eyelid droops	3 (20)
Diarrhea	2 (13.3)
Arm or leg weakness	2 (13.3)
Pain disturbs sleep, *n* (%)	
Never	0 (0)
Occasionally	8 (53.3)
Often	7 (46.7)
Affected quality of life, *n* (%)	
Not at all	0 (0)
Moderately	3 (20)
Extremely	12 (80)

ON, occipital neuralgia; IQR, interquartile range; SD, standard deviation.

Preoperatively, all patients trialed at least three different pharmacologic classes of preventive medications, among which three patients (20%) were on no preventative medications prior to surgery due to lack of efficacy and/or side effects. An average of 11.7 (±9) BTX injection cycles were administered per patient. At the time of surgical screening, BTX had been discontinued in one (6.7%) patient and had reduced effectiveness over time in 6 patients (40%). An average of 10.2 (±5.8) NB injection cycles were performed per patient. Among the study patients, the maximum duration of relief after an NB was 3 months, but unfortunately that duration of benefit was not sustained with subsequent blocks, which prompted surgical evaluation. One (6.7%) patient had undergone previous radiofrequency ablation (RFA), which provided only temporary relief. Twelve (80%) patients reported having undergone physical therapy. Other treatment modalities included acupuncture, chiropractic therapy, and meditation with limited improvement. The average duration of medical management by the referring headache specialist before referral to the plastic surgeon for nerve decompression surgery was 3.9 (±2.8) years.

**Figure 1:**
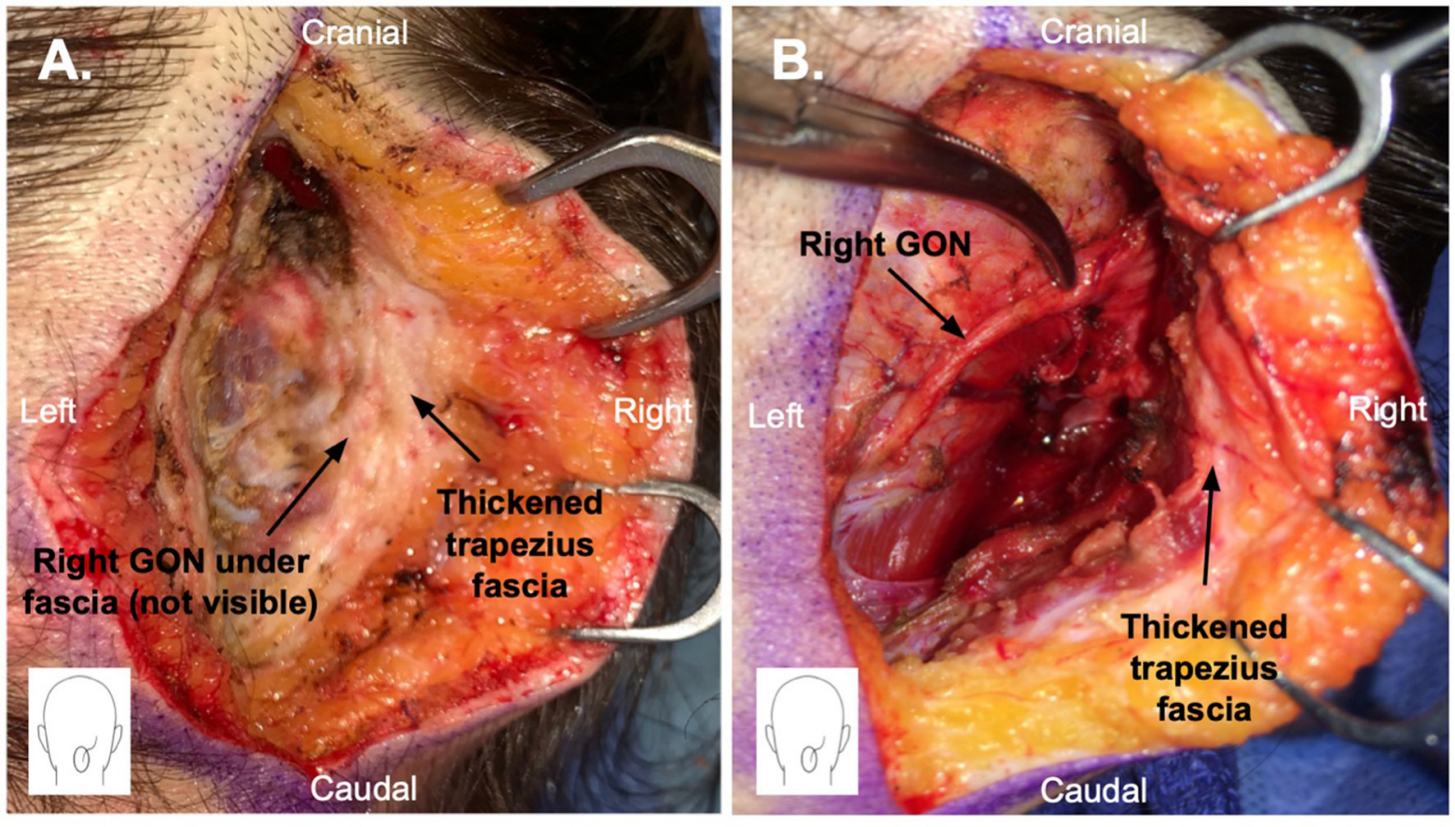
Greater occipital nerve (GON) decompression surgery through a 5 cm vertical midline incision in the posterior scalp. **(A)** Thickened trapezius fascia is shown. The GON is not yet visible as it is stuck within the thickened fascia. **(B)** Thickened trapezius fascia has been elevated, and the GON has been dissected free.

Patients underwent bilateral (13 patients, 86.7%) or unilateral (2 patients, 13.3%) greater occipital nerve (GON) decompression. A thickened trapezius fascia overlying the GON was observed intraoperatively in 12 (80%) patients (Figure 1). Any contact between the GON and the occipital artery was observed in 7 (46.7%) patients, and the contact was extensive in most cases (57%). Simultaneous LON decompression was performed in 9 (60%) patients and TON decompression in 3 (13.3%) patients. The decision to include LON and TON decompression was based on history, physical examination, NB response, and/or intraoperative findings. The mean postoperative follow-up period was 16.8 (±9.7) months.

At 12 months postoperatively, median lancinating ON headache frequency was 5 (0-16) days/month (*p* < 0.01), intensity was 4 (0–6) (*p* < 0.01), and duration was 10 (0–24) h (*p* < 0.01). In terms of lancinating ON pain, the median patient-reported percent resolution was 80% (70%−85%). In 4 (26.7%) patients, 100% resolution was reported, in 5 (33.3%) patients, ≥80% resolution was reported in 4 (26.7%) patients, ≥50% resolution was reported, and in 2 (13.3%) patients, ≤ 20% resolution was reported. All patients reported a reduction or discontinuation of at least one class of medications. In 11 (73.3%) patients, medications were reported to be more effective after surgery as compared to preoperatively. All patients reported increased effectiveness and/or reduced frequency of needed occipital NB or BTX injections after surgery. One patient completely stopped all medications, BTX, and NB.

All patients reported that surgery had helped relieve their concomitant headache disorders, which was in most cases migraine. However, although improved after surgery, patients still continued to experience migraine symptoms such as fronto-orbital pain, difficulty concentrating, photophobia, phonophobia, and nausea during headache attacks. Postoperative outcomes are summarized in Table 4.

**Table 4:** Postoperative outcomes.

**Variable**	**Patients (*n* = 15)**
ON pain characteristics, median (IQR)	
Frequency, days per month	5 (0–16)
Intensity, scale 1-10	4 (0–6)
Duration, hours	10 (0–24)
Percent improvement in ON pain, *n* (%)	
100%	4 (26.7)
≥80%	5 (33.3)
≥50%	4 (26.7)
≤ 20%	2 (13.3)
Change in medications, *n* (%)	15 (100)
Reduction in quantity per month	15 (100)
Discontinuation of at least 1 medication class^*^	10 (66.7)
Discontinuation of opioid-based medication	2 (50.0)
Discontinuation of all medication	1 (6.7)
Improved effectiveness	11 (73.3)
Change in nerve blocks, *n* (%)	13 (87.7)
Reduction in frequency/increased effect	9 (60.0)
Discontinuation	4 (26.7)
Change in BTX, *n* (%)	10 (66.7)
Reduction in frequency/increased effect	9 (60.0)
Discontinuation	1 (6.7)

BTX, Onabotulinum toxin A; ON, occipital neuralgia; IQR, interquartile range.

^*^Including anti-inflammatory, abortive, preventive, anticonvulsant, opioid or antiemetic.

Among the two patients who reported < 50% resolution, the first was diagnosed with ON, persistent posttraumatic headache with migrainous features, and trigeminal neuralgia following multiple direct strikes to the head and concussions. She was known to have intractable head pain necessitating repeated ED visits. She responded to NB with some relief (< 50%) for < 48 h at the time of surgical evaluation. After bilateral GON decompression, the patient initially reported 95% relief at 3 months postoperatively, but her ON headache returned predominantly on the right side, reporting an overall 0% resolution of her ON headaches at 12 months. The patient did, however, report discontinuing antiemetics and increased efficacy of NB. Reoperation was performed with GON transection and nerve end reconstruction on the side of the predominant ON headache and repeat GON decompression on the other side. At 12 months after reoperation, the patient reported a 95% resolution of her occipital pain.

The second patient who reported < 50% resolution was diagnosed with ON, chronic migraine, and cervical radiculopathy status postmicrodiscectomy. The onset of ON was with no known initiating factor or history of head or neck injury. The patient reported intractable pain with multiple ED visits. At the time of surgical evaluation, NB provided significant relief (>50%) but for < 48 h. After 32 years since ON onset, the patient underwent unilateral GON, LON, and TON decompression. At 12 months postoperatively, she reported only a 20% resolution of ON headache with a reduction in the intensity of her ON headaches but not in frequency or duration. Postoperatively, she reported discontinuation of pregabalin, reductions in opioid and antiemetics, and better efficacy of medications. She did not endorse any changes in the use of BTX and NB. This patient did not undergo reoperation; however, given her diffuse body pain, she was seen in a peripheral nerve clinic and was diagnosed with small fiber neuropathy. During 2023 follow-up appointments, this patient's ON and migraine have been under relatively good control with 10 or fewer severe headache days per month as compared to her small fiber neuropathy pain, which has been daily and disabling.

## Discussion

This case series (1) described the clinical features of patients with refractory ON amenable to surgical treatment, (2) demonstrated that all patients benefited from occipital nerve decompression surgery to varying degrees, and (3) suggests that the collaborative selection criteria employed by neurologist and surgeon may be replicable in clinical practice.

### Clinical features of patients with refractory ON amenable to surgical treatment

The clinical diagnosis of ON is challenging, and its true prevalence is unknown. Although previous studies have reported on the clinical features of ON, few have described the features of chronic refractory cases potentially amenable to surgical decompression (4, 6, 31–36). In this case series, patients with chronic refractory ON exhibited all symptoms described by the ICDH-3 diagnostic criteria but also reported additional symptoms that have not been significantly reported in the literature.

First, we observed a higher prevalence of bilateral ON headache compared to unilateral ON headache. While the ICDH-3 acknowledges that ON can manifest as both unilateral and bilateral, previous studies have reported a predominance of unilateral ON, which contrasts with the results from our series (33, 34, 36, 37). This increased incidence of bilateral ON headache may be attributed to the chronicity of ON in our series or could signify the underlying pathophysiology involving diffuse nuchal myofascial hypertrophy following head or neck injury (22).

Second, patients frequently presented with pain radiating from the occipital to the fronto-orbital regions, which can be clearly visualized with the use of pain drawings. Such distribution of pain has been documented by others and is thought to be due to either extracranial anastomoses of the occipital nerves with trigeminal nerves or due to referred pain mechanisms at the level of the trigeminocervical complex (34, 36). Since frontal pain may also manifest in other headache disorders, its presence may contribute to instances of ON misdiagnosis or underdiagnosis.

Third, in addition to experiencing short paroxysms of lancinating pain, shooting, stabbing, or sharp in quality and lasting from a few seconds to minutes, patients in our series presented with constant achy pain in the same distribution. In clinical practice, this is commonly seen with ON as well as in other paroxysmal pain disorders such as sciatica and trigeminal neuralgia. This is why the ICHD-3 diagnostic criteria for trigeminal neuralgia include subtypes of the purely lancinating form and the form that has a steady baseline pain in the same distribution as the superimposed lancinating pain (3). Aching, pressure, pounding, or throbbing sensations, which were at times extra-occipital, may have been a manifestation of coexisting chronic migraine or other headache disorder. The protracted ON headache experienced in these patients could be due to the chronic nature of the condition after a duration of symptoms on average 19.9 years and/or central sensitization (38). Alternatively, it could be indicative of a constant compression point by the surrounding tissue. In contrast, episodic ON would be less likely to be associated with anatomical compression (22).

Furthermore, this study highlighted the high prevalence of coexisting headache disorders among patients with ON. The coexistence of ON with other primary headache diagnoses such as migraine may be an underrecognized phenomenon and has been reported to be seen in up to 25% of patients presenting to a headache specialty clinic (4). In our case series, all patients had coexisting headache disorders. However, distinguishing multiple headache disorders may be challenging due to a significant overlap of clinical features between ON, migraine, cervicogenic headache, cluster headache, and tension headache (3). Nevertheless, distinguishing ON from other headache diagnoses is important because the treatment is vastly different. Therefore, patients presenting with headache disorders should be screened for ON.

### All selected patients benefitted from nerve decompression surgery

We found that all patients in our series benefited from nerve decompression surgery to varying degrees. Resolution of lancinating ON headache was evidenced by reductions in frequency, intensity, and duration of headaches, as well as significant reductions and/or increased effectiveness of medications, BTX, and NB at 12 months.

Nerve decompression surgery has been previously shown to be an effective treatment option for refractory ON (24–29, 39–43). Studies that have analyzed outcomes following nerve decompression surgery for headaches have mostly reported reductions in headache intensity of −4 points and reductions in headache frequency of −7 and −20 headache days/month, which conform with our findings (25, 26, 29, 39, 44). Less has been reported on changes in the duration of headaches, but existing reports have found significant reductions similar to our study (45). Moreover, the success rate (>50% resolution in ON headache) following occipital nerve decompression surgery has been reported to be approximately 80% (range 68–95%), in line with our results (26, 42–44, 46). Furthermore, the decrease in postoperative daily medication use after nerve decompression surgery corroborates with the findings of several other studies (43, 44, 47). The improvement in postoperative efficacy of medications, BTX, and NB suggests that ON may act as a barrier to response to these therapies, and nerve decompression surgery likely indirectly improves coexisting headache disorders.

It could be posited that, given the substantial disease burden and the treatment-resistant nature of all patients in this series prior to surgery, this particular cohort of ON patients may exhibit a higher degree of surgical failure as compared to patients in other studies. However, our findings did not align with this expectation. Not only did patients experience resolution of ON (both lancinating and baseline occipital pain) but patients also reported reductions in comorbid headache disorders, most frequently migraine.

In our series, two patients reported < 50% resolution of ON headache at 12 months postoperatively. However, as illustrated in the first patient, revision surgery may be needed at times to achieve >50% resolution. Both cases prompt us to better understand risk factors associated with poor outcomes or reoperation.

Risk factors have been previously described in the literature and include poor NB response, atypical pain drawings, RFA, and cervical spine disorders. While most patients in our series had days to weeks of ON headache relief with NB at the time of screening, both patients had the lowest duration lasting < 48 h. It has been reported that a NB response of < 24 h is associated with worse outcomes following nerve decompression surgery (48). An absence of NB response would conflict with the diagnostic criteria of ON set forth by the ICDH-3 and can be a relative contraindication to surgery. Atypical pain sketches have also been shown to predict poor surgical success, while a history of RFA and cervical spine disorders have been shown to be associated with a higher number of revision surgeries and nerve transections to achieve acceptable outcomes (49–51). Suboptimal outcomes in these patients may also be due to incomplete decompression during primary surgery and/or subsequent scar tissue formation. A better understanding of risk factors for poor outcomes will aid in refining patient selection criteria.

### Collaborative patient selection criteria for nerve decompression surgery

The favorable outcomes observed following occipital nerve decompression surgery in our cohort suggest that the collaborative selection criteria employed in this study could be replicable in clinical practice. The listing of these criteria is not arranged in a temporal order and need not be considered in any particular order without overlap. For example, during the initial visit, the patient may receive a NB, a prescription for gabapentin that can be initiated by the patient depending on NB response, as well as a referral for physical therapy.

The selection criteria for surgical candidacy, which involve assessments by both a headache specialist and a surgeon, highlight the importance of a multidisciplinary approach for optimal treatment of patients with refractory ON. While the headache specialist/neurologist plays a critical role in the diagnosis of refractory ON and in providing the best preoperative management before referral, the surgeon evaluates potential compression sites that can be surgically addressed to alleviate nerve compression symptoms. This relationship is bidirectional, as the surgeon is to refer patients, especially those who are self-referred or referred by their primary care physician, to a headache specialist/neurologist to confirm the diagnosis of ON and ensure that the patient has failed the best medical/interventional management before proceeding with surgery. These multidisciplinary selection criteria may guide the development of future treatment algorithms for patients with refractory ON.

### Limitations

This study was limited by the lack of a control group, challenging the establishment of a cause-and-effect relationship between the surgical intervention and the observed outcomes. Although there are several studies in the literature highlighting the significant morbidity associated with ON as well as numerous studies reporting on the effectiveness of nerve decompression surgery, the risk of a placebo effect is particularly high in headache patients and should be acknowledged.

## Conclusion

In conclusion, ON can cause disabling headaches and may be highly underdiagnosed.

The frequent coexistence of ON with other headache disorders likely contributes to its underdiagnosis. Therefore, screening for ON should be considered among other headache patients to improve ON diagnosis and treatment.

Furthermore, we demonstrated that for patients with ON that is refractory to conservative therapies, occipital nerve decompression surgery can be an effective treatment, improving lancinating ON pain as well as the effectiveness of medications, NB and BTX. Additionally, surgical treatment of ON was able to improve coexisting headache disorders such as migraines.

Therefore, the collaborative selection criteria employed in this study neurologist/headache specialist and surgeon may be replicable in clinical practice. Future efforts should be made to include and define occipital nerve decompression surgery within current and future ON treatment algorithms.

## Data availability statement

The raw data supporting the conclusions of this article will be made available by the authors, without undue reservation.

## Ethics statement

The studies involving humans were approved by Massachusetts General Hospital, Boston. The studies were conducted in accordance with the local legislation and institutional requirements. The participants provided their written informed consent to participate in this study. Written informed consent was obtained from the individual(s) for the publication of any potentially identifiable images or data included in this article.

## Author contributions

WA: Conceptualization, Methodology, Project administration, Resources, Validation, Writing—review & editing. KR: Data curation, Formal analysis, Writing—original draft. KP: Data curation, Writing—original draft. MH: Data curation, Writing—original draft. LG: Conceptualization, Methodology, Project administration, Supervision, Validation, Writing—review & editing. PM: Conceptualization, Methodology, Project administration, Supervision, Validation, Writing—review & editing.
